# Place Matters: Understanding Geographic Influences on Youth Not in Education, Employment, or Training—A Scoping Review

**DOI:** 10.1002/jad.12461

**Published:** 2025-01-09

**Authors:** Victoria Lindblad, Rolf L. Lund, Pernille Skou Gaardsted, Line Elise Møller Hansen, Fie Falk Lauritzen, Dorte Melgaard

**Affiliations:** ^1^ Department of Gynecology, Pregnancy and Childbirth North Denmark Regional Hospital Hjoerring Denmark; ^2^ Department of Sociology and Social Work Aalborg University Aalborg Denmark; ^3^ Medical Library Aalborg University Hospital Aalborg Denmark; ^4^ Department of Acute Medicine and Trauma Care Aalborg University Hospital Aalborg Denmark; ^5^ Faculty of Clinical Medicine Aalborg University Aalborg Denmark

**Keywords:** education, geographical risk factors, NEET, neighborhood, systematic review, unemployment

## Abstract

**Introduction:**

Youth aged 15–29 who are not engaged in education, employment, or training (NEET) represent a critical concern within the European Union (EU).

**Aim:**

This review aims to ascertain whether existing studies address the impact of living in either rural or urban settings, or in specific types of neighborhoods, on the likelihood of young European individuals falling into NEET status.

**Methods:**

On February 21, 2023, and subsequently updated on January 15, 2024, a thorough literature search was carried out across four major databases to compile relevant studies.

**Results:**

From an initial pool of 33,314 articles, 11 studies were deemed relevant for this review involving over 786,399 participants. The analysis revealed that residing in disadvantaged neighborhoods, characterized by significant crime rates and unemployment levels surpassing national averages, correlates strongly with an increased incidence of NEET status among youth. Notably, impoverished areas with a high presence of visible minorities were associated with higher rates of school dropout or unemployment. Furthermore, the conditions of the local labor market were found to notably affect dropout rates from secondary schools, especially in urban centers. Whereas rural areas exhibited elevated unemployment rates among the youth.

**Conclusions:**

This review underscores the need for targeted policies that address geographical disparities in NEET status by tailoring interventions to urban, rural, and neighborhood‐specific contexts. Policymakers should focus on localized support programs and integrate geographical factors into strategic planning to ensure equitable opportunities for all youth.

## 1 Introduction

In recent decades, the issue of young individuals not engaged in education or employment has had significant attention from policymakers globally, prompting various intervention initiatives amidst high political expectations. The term “NEETs” refers to young people aged 15–29 years and not engaged in Education, Employment, or Training (OECD 2024). The concept of NEET emerged in UK policy documents in the late 1990s and was officially adopted into the European Union's policy framework in 2010 (Redmond and McFadden 2023). During that period, the European Commission Employment Committee (EMCO) established a standardized definition and methodology for NEET to facilitate monitoring youth disadvantage across Europe. Recently, in alignment with the 2020 Reinforced Youth Guarantee, the focus of NEET analysis within the EU has been on individuals aged 15–29 (European Parliament 2017; Redmond and McFadden 2023). The average number NEETs in the Organization for Economic Cooperation and Development (OECD) European countries was in 2023 11.2% in the European countries, and ranging from 4.8% in the Netherlands to 19.3% in Romania (Eurostat 2024b). Although the NEET term is European in origin, the issue of young people disconnected from education and employment is a global concern. In the United States, this group is known as “disconnected youth” or “opportunity youth,” in Latin America, they are referred to as “NiNis,” and in Japan they are called “freeters” (Kevelson et al. 2020). Other synonyms for this group in the literature include unengaged youth, out‐of school youth, economically inactive youth, and marginalized youth. The young in the NEET group often have lower levels of education and limited work experience and skills. They may also face physical and mental health issues and unstable marital statuses. Additionally, they are frequently impacted by poverty, social inequalities, suboptimal living conditions, and their parents' economic situations, including income, education, and job status (Rahmani and Groot 2023).

In light of evolving global economic landscapes and the acute aftermath of the COVID‐19 pandemic, the urgency to address the NEET phenomenon has never been more pronounced (OECD 2020). These shifts, alongside technological advancements, have not only transformed the nature of work but have also deepened existing inequalities, making the path from education to employment even more precarious for Europe's youth (International Labor Organization 2020; OECD 2020). The sociopolitical ramifications of a disengaged youth population—ranging from social unrest to economic strain—underscore the necessity for nuanced, evidence‐based policy interventions (Furlong 2016; Maguire 2015). Furthermore, geographical disparities, characterized by the rural‐urban divide, neighborhood effects, and unequal access to resources, highlight the complex, systemic nature of youth unemployment, and education disengagement. Despite various policy initiatives at national and EU levels aimed at mitigating youth unemployment, there remains a significant gap in addressing the intricate geographical determinants that contribute to the NEET status (Euorpean Comission 2020; Rodríguez‐Pose and Tselios 2019).

It is important to gain insight into which young people are particularly challenged in the school system and in the transition to the labor market to be able to launch early efforts to support the young. Research consistently shows that certain demographic groups face a heightened risk of becoming NEETs compared to others (Gariépy et al. 2022; Rahmani, Groot, and Rahmani 2024; Tayfur et al. 2022).

Limited focus is regarding how geographical factors such as residing in rural versus urban areas and the specific neighborhood one lives in influence the risk of becoming NEET, and none of the existing reviews include or focus on geographic factors.

A report based on the Scottish Longitudinal Study Census data highlights a clear link between geographical factors and NEET status, with 2015 data showing a significant rise in NEET rates in areas of higher deprivation and a tendency for these rates to be higher in urban settings (Feng et al. 2015). In 2023, data from the European Union indicated that the proportion of young people (aged 15–29) who were NEETs was lowest in cities (10.3%), with slightly higher rates in towns and suburbs (11.7%) and rural areas (12.3%) (Eurostat 2024b). This pattern of lower NEET rates in urban areas compared to rural and suburban regions was consistent across 17 EU Member States.

However, this data may not fully represent the broader European context, which includes over 40 countries with diverse socioeconomic landscapes. We believe that variations in the socioeconomic landscape matter and aim to uncover a more nuanced understanding of how location impacts young people's access to education and employment opportunities. A deeper investigation into how geographical factors influence the likelihood of young people becoming NEET across different regions would provide a more detailed and accurate understanding of how location affects their educational and economic opportunities. By thoroughly exploring the complexities of young people's lives and the factors that shape their experiences, we can ensure that public policies and services are more effectively tailored to address these challenges and create meaningful change.

To gain an overview of the existing quantitative literature we chose to conduct a scoping review and to lay a solid foundation for understanding the current knowledge of the topic and guiding future research. We focused on synthesizing a broad range of research to provide an overview of the topic rather than evaluating the rigor of individual studies, and therefore, we did not assess the quality of the included studies.

This review aims to unpack the geographical factors influencing NEET status among European youth. To achieve this, we conducted a comprehensive systematic literature search to identify geographical factors impacting youth education and employment trajectories from 1980 onward. We will first outline the methods used in the literature search, followed by a presentation of the search results. Finally, we will discuss the perspectives on geographical factors explored in the evidence and their implications for policymakers. By doing so, it not only fills a critical gap in the existing literature but also lays down a foundation for developing more targeted, effective interventions that consider the nuanced sociogeographical dynamics at play. Such an approach is crucial for crafting policies that are not only responsive to the current economic and social landscape but also resilient to future shifts (Cuervo and Wyn 2014; Wong, Zimmerman, and Parker 2010).

## 2 Methods

The article employed the PRISMA‐ScR Checklist for structuring, as outlined by Tricco et al. (2018). This scoping review is part of a broader report comprising four reviews that investigate geographical, physical, psychological, and social factors contributing to young individuals not completing education or entering the labor market, thereby becoming part of the NEET group. A comprehensive systematic literature search, detailed below, serves as the foundation for these four reviews.

### 2.1 Eligibility Criteria

To establish clear inclusion criteria for this literature review, we employed the PCC (participants, concept, and context) approach (Pollock et al. 2023). Participants meeting the inclusion criteria were young individuals aged 15–29 years who were classified as NEETs according to Eurostat's definition (OECD 2024). The concept of NEET encompassed young people who were not currently employed and had never entered the workforce after completing compulsory education. This included individuals who had not started secondary school within the preceding four weeks or had not completed secondary school, and who were not working or undergoing training. The data used in the study had to be collected in a European country after 1980. By concentration on more recent studies, we ensure that the findings are relevant to current contexts and reflective of contemporary challenges faced by NEET youths. We have included 43 countries on the European continent (European Commission 2024). We have chosen to include only quantitative literature, as our aim is to synthesize the data to gain an overview of the measurable outcomes, which requires a focus on objective data. Only quantitative data from primary research was considered, with mixed methods studies requiring distinguishable quantitative data. Articles written in English, German, Danish, Norwegian, and Swedish were included to ensure a comprehensive and diverse review of the NEET concept. Other languages were not included due to limitations in resources for translation and review. Experimental studies were excluded, as the focus was on naturally occurring risk factors.

### 2.2 Information Sources and Search Strategy

Medline, Embase, PsycINFO, and Scopus were systematically searched on February 21, 2023, and updated on January 15, 2024. The search consisted of two aspects, the population, and the outcome. The population was young people (and synonyms) and the outcome was the NEET group (and synonyms). No additional limitations were made as the goal was to find all risk factors for becoming NEET. A combination of search techniques was used, including proximity operators, controlled vocabulary thesaurus terms for example, medical subject headings (MeSH), and a free text search of all the synonyms and variations of the keyword. The search terms were combined with Boolean operators AND or OR (Grewal, Kataria, and Dhawan 2016). Finally, the searches were supplemented with forward citation searching in Citationchaser (Haddaway, Grainger, and Gray 2021). The search strategies were peer reviewed by a specialist medical librarian outside the author group and the search strategies from all databases are shown in Aappendix S1.

### 2.3 Selection Process and Data Extraction

The selection process was divided into two rounds. First, all the authors independently screened the studies on title and abstract in Covidence excluding all studies that were not conducted in a European country. The first round only required an exclusion decision from one author. Second, the authors independently screened the records in Covidence according to the inclusion criteria. The second round required two authors to decide whether the studies should be included or excluded based on the title and abstract. Disagreements in the screening process were discussed and resolved by the consensus of all authors. Authors V. L. and D. M. cooperated on the full‐text screening. Disagreements were solved by consensus within the author group. Information about author(s), year of publication, title, aim(s), participants (number, age, and nationality), study design, and main findings were extracted from studies and charted.

We used an inductive approach to synthesize the findings through collaboration among the authors, systematically identifying patterns and themes across the included studies in an iterative process. This method allowed the data to guide the synthesis, with a focus on specific geographical issues under investigation. When multiple studies addressed the same factors, we summarized and presented the results collectively in Section 3 (Pollock et al. 2023).

## 3 Results

**Figure 1:**
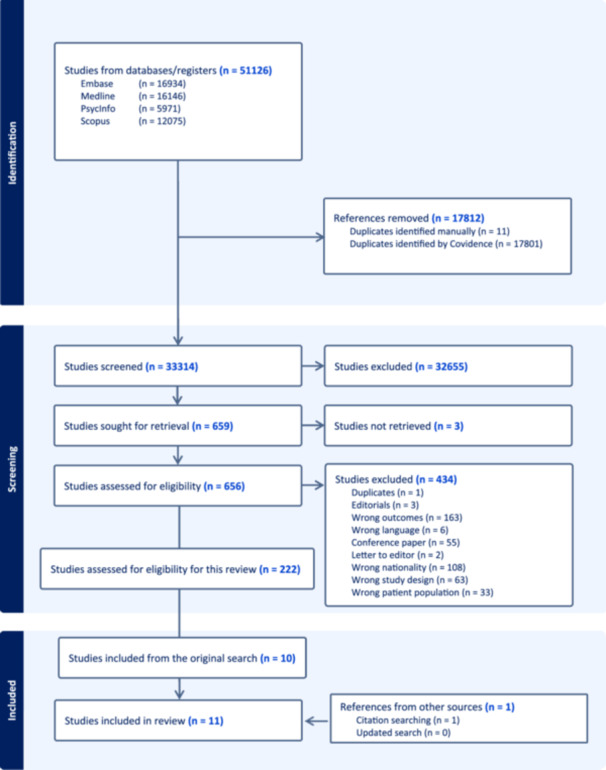
Flow diagram of screening process.

The authors conducted a systematic and comprehensive literature search according to the methods described above to identify relevant articles. As illustrated in Figure 1 the search strategy identified 51,126 studies. After removing duplicates 33,314 studies were screened independently based on title and abstract, and 32,655 were irrelevant. A total of 659 studies were screened in full text and 434 studies were excluded. Three studies could not be retrieved. Thus, 222 studies were included, and 10 studies were included from the original search. Only 1 additional study was added after the citation search making a total of 11 studies included in this review.

All 11 studies were published after 2012. The study designs comprised cohort studies, except for one cross‐sectional study (von Simson 2015). The combined total of participants across all studies was 786,399. In addition, one study examined all individuals born in 1985 and residing in a metropolitan area in Sweden between 2001 and 2006.

The participants were drawn from Scandinavian countries in six studies with three from Sweden (Brännström and Rojas 2012; Gustafsson, Katz, and Österberg 2017; Helgesson et al. 2018) and three from Norway (Brattbakk and Wessel 2013; Myhr et al. 2017; von Simson 2015), three from the United Kingdom (Goldman‐Mellor et al. 2016; Magdalene Karyda and Jenkins 2018; Magdalini Karyda 2020), and one each from Latvia (Grinevica and Rivza 2015) and Finland (Lallukka et al. 2019). There is significant heterogeneity among the studies, not only in terms of the countries they originate from but also in the topics they cover, such as the urban‐rural divide, neighborhood effects, local labor market conditions, and sociogeographic dynamic.

In metropolitan neighborhoods, a labor market failure among longer educated parents and neighbors was associated with young people not completing upper secondary high school and subsequently becoming NEET (17% males and 14% females) (Gustafsson, Katz, and Österberg 2017). Additionally, one other study found that urban residence was associated with school completion, but only among tertiary‐educated individuals (OR 1.19, CI: 1.06–1.32) (Myhr et al. 2017), while another study found that rural regions had the highest youth unemployment rates (respectively 47.2% and 30%) (Grinevica and Rivza 2015). Living in rural areas increased the likelihood of labor‐market marginalization among young people with mental disorders. In medium‐sized cities, the odds ratio (OR) for high unemployment trajectories was 2.01 (CI: 1.71–2.36), and in small cities/villages, it was 2.09 (CI: 1.73–2.52) (Helgesson et al. 2018).

As documented in Table 1 living in a disadvantaged neighborhood increases the likelihood of young people becoming NEET. Disadvantaged neighborhoods were associated with young being NEET in six studies. These neighborhoods were characterized by various factors, including high crime levels (Magdalene Karyda and Jenkins 2018; Magdalini Karyda 2020) and municipalities with higher rates of unemployment than the national median (≥ 10.7%) (Men aOR 1.58, CI: 1.42–1.76 and women aOR 1.77, CI: 1.52–2.06) (Lallukka et al. 2019). Two studies also found that living in poor, deprived neighborhoods (41.5%, *p *< 0.001) or neighborhoods with visible minorities populations increased the likelihood of young people dropping out of school or becoming unemployed (RRR = 1.34–1.76, depending on the type of neighborhood) (Brännström and Rojas 2012; Goldman‐Mellor et al. 2016). A study found that at age 25, youth unemployment was significantly influenced by the share of unemployed (coefficient = 0.115, SE = 0.058), low‐educated (coefficient = 0.022, SE = 0.011), and low‐income individuals (coefficient = 0.021, SE = 0.010) in the neighborhood (Brattbakk and Wessel 2013). Additionally, the share of people on social assistance (coefficient = 0.038, SE = 0.015), receiving transitional benefits for single parents (coefficient = 0.048, SE = 0.022), or rehabilitation benefits (coefficient = 0.132, SE = 0.054) also had an effect. By age 29, only the share of individuals on disability pensions (coefficient = 0.156, SE = 0.069) influenced youth unemployment (Brattbakk and Wessel 2013).

**Table 1:** Summary of study details and population (sorted after publication year).

References	Title	Aim(s) of the study	Participants; number, age, and nationality	Study design	Main findings
Karyda (2020)	The influence of neighborhood crime on young people becoming not in education, employment, or training	Does social disorganization, understood as the level of crime experienced in the neighborhood influence young people's educational and employment outcomes?	8931 young people in 1989/90. Data were collected when the young people were 19 years—United Kingdom.	Cohort	Living in a disadvantaged neighborhood increases the probability of young people experiencing Not in Education, Employment or Training (NEET) status at ages 16–19. There is a dose‐response relationship, with higher crime rates in the neighborhood linked to a greater risk of becoming NEET. Living in a high‐crime area was associated with an 80% higher likelihood of entering the NEET group compared to those in low‐crime areas.
Lallukka et al. (2019)	Determinants of long‐term unemployment in early adulthood: A Finnish birth cohort study	Social and health‐related determinants to long‐term unemployment during early working life among young adults	46,521 adolescents at the age of 25–28 years—Finland	Cohort	The risks for unemployment were observed for low and high unemployment municipalities, but they were generally higher for those living in municipalities with unemployment rates higher than the country median level (≥ 10.7%). Men had an adjusted odds ratio (aOR) of 1.58 [1.42, 1.76] for long‐term unemployment in municipalities with high unemployment rates, while women had a higher aOR of 1.77 [1.52, 2.06].
Helgesson et al. (2018)	Trajectories of work disability and unemployment among young adults with common mental disorders	To contribute to research into neighborhood effects on young adult outcomes	7245 19–30 years with an incident diagnosis of a common mental disorder in the year 2007, and a matched comparison group of 7245 individuals without mental disorders—Sweden	Cohort	Living in rural areas was factor that increased the probability for labor‐market marginalization among young people with common mental disorders. In medium‐sized cities, the odds ratio (OR) for being in the high‐increase unemployment trajectory group was 2.01 [1.71, 2.36] compared to the constant low unemployment trajectory group. For small cities and villages, the OR was 2.09 [1.73, 2.52].
Karyda and Jenkins (2018)	Disadvantaged neighborhoods and young people not in education, employment, or training at the ages of 18–19 in England	The effects of neighborhood context on young people who experience NEET status	2436 adolescents aged 19 years—United Kingdom	Cohort	There is a significantly higher probability (85%, *p *< 0.001) for young people who live in high‐crime areas to become NEETs compared to those living in less‐deprived areas
Gustafsson, Katz, and Österberg (2017)	Why do some young adults not graduate from upper‐secondary school? On the importance of signals of labor market failure	Investigating factors associated with not graduating from upper‐secondary school and exploring the variation in the proportion of 21‐year‐olds without upper‐secondary school education across metropolitan neighborhoods	All individuals born in 1985 and living in a metropolitan area in 2001–2006, 16–21 years old—Sweden	Cohort	The labor market success or failure of relevant adults surrounding the young person is associated with 17% of young males and 14% of females not completing upper‐secondary school. As the share of unemployed highly educated adults in a neighborhood increases from 0% to 4%, the risk for young people rises sharply but slows at higher levels. For young women, the risk increases significantly once the share reaches 8%, suggesting a threshold effect. For young men, the effect is weaker, with only a slight increase at 11%. In short, the negative impact on educational engagement is stronger for young women when the share exceeds 8%.
Myhr et al. (2017)	Do family and neighborhood matter in secondary school completion? A multilevel study of determinants and their interactions in a life‐course perspective	Investigate the variation and clustering of completion of upper secondary education in families and neighborhoods	107,003 aged 21–27 years in 2010—Norway	Cohort	Urban residence was associated with school completion, but only among the tertiary educated (OR 1.19, CI 1.06–1.32). Urban settlement is linked to lower odds of completing secondary education (OR = 0.94), but this association disappears after adjusting for parental employment and poverty. However, when considering the interaction between urban residence and parental education, the effect becomes significant, suggesting that the impact of urban living varies depending on the education level of the parents.
Goldman‐Mellor et al. (2016)	Committed to work but vulnerable: Self‐perceptions and mental health in NEET 18‐year‐olds from a contemporary British cohort	The impact of family structure and neighborhood on school completion	2232 twins born in 1994–1995 data collected at the age of 18 years—United Kingdom	Cohort	NEET youths were significantly more likely than their non‐NEET peers to come from poor neighborhoods. A significantly lower proportion of NEET youth (12.2%) live in wealthy neighborhoods compared to non‐NEET youth (27.9%, *p* < 0.001). There is no significant difference in the proportion of NEET youth living in urban prosperity (3.5% vs. 5.0%, *p* = 0.450) or comfortably off neighborhoods (24.5% vs. 30.2%). However, a higher proportion of NEET youth live in moderate means neighborhoods (18.3% vs. 13.2%), though this difference is not statistically significant. A significantly higher proportion of NEET youth (41.5%) live in hard‐pressed neighborhoods compared to non‐NEET youth (23.8%, *p* < 0.001), highlighting a strong link between neighborhood deprivation and NEET status.
Grinevica and Rivza (2015)	Analysis of youth integration into the labor market by the Quintuple helix model in Latvia's regions	Comparing four types of work‐related self‐perceptions, as well as vulnerability to mental health and substance abuse problems, among youths not in education, employment, or training (NEET) and among their peers	27,100 15–24 years old—Latvia	Cohort	Youth unemployment is highest in the rural regions, with the Latgale region experiencing the highest rate at 47.2%, followed by Kurzeme with a rate of 30%
von Simson (2015)	Explaining upper secondary school dropout. New evidence on the role of local labor markets.	Analyzing youths' integration into the labor market	492,078 participants 16–24 years—Norway	Cross‐sectional	The results show that local labor market conditions play a substantial role in individual dropout decisions with elasticities ranging from 0.1 to 0.3. The opportunity cost of schooling seems to weigh more in the dropout decision for youth than the expected returns. However, the results are highly sensitive to the choice of local labor market indicator. Local labor market conditions negatively influenced individuals' decision to dropout of secondary school (OR: 0.133, *p* > 0.01). Better labor market conditions significantly decrease the likelihood of young people (ages 16–24) dropping out of academic programs, with an odds ratio (OR) of 0.249 for girls and 0.281 for boys.
Brattbakk and Wessel (2013)	Long‐term neighborhood effects on education, income, and employment among adolescents in Oslo	To assess the impact of local labor market conditions on upper secondary school dropout among youth, with a specific focus on the desire to work as a potential motivation for leaving education early	5516 adolescents aged 21, 25, and 29 years—Norway	Cohort	Neighborhood deprivation during adolescence has a small but significant effect on educational achievement and income later in life. The concentration of welfare recipients in the neighborhood is strongly associated with socioeconomic status, highlighting the importance of social value and social participation. Neighborhood unemployment significantly affects youth unemployment at age 21 (coefficient = 0.115, SE = 0.058). The share of low‐education individuals (coefficient = 0.022, SE = 0.011) and low‐income individuals (coefficient = 0.021, SE = 0.010) in the neighborhood also have significant effects. At age 25, the neighborhood share of individuals on social assistance (coefficient = 0.038, SE = 0.015), transitional benefits for single parents (coefficient = 0.048, SE = 0.022), and rehabilitation benefits (coefficient = 0.132, SE = 0.054) are significant predictors of youth unemployment. At age 29, only the share of people receiving disability pensions impacts youth unemployment (coefficient = 0.156, SE = 0.069). All results are significant at the 5% level (*p* < 0.05).
Brännström and Rojas (2012)	Rethinking the long‐term consequences of growing up in a disadvantaged neighborhood: Lessons from Sweden	To investigate the impact of neighborhood deprivation on educational achievement, income, and unemployment, determine if the effect of neighborhood influence attenuates over time, and assess the extent to which different neighborhood indicators yield a similar outcome	80,092 adolescents (48,6% female), 16–27 years—Sweden	Cohort	Growing up in a disadvantaged neighborhood increases the risk of experiencing more unemployment, having less education, and receiving more social assistance compared to similar young people from more affluent neighborhoods. Young people from neighborhoods with higher concentrations of resource‐poor groups and visible minorities were less likely to be found in the cluster with comparably more education. Young people from more disadvantaged neighborhoods were not more likely to figure in the comparably less education cluster or the comparably more unemployment cluster. Youth from neighborhoods with high concentrations of resource‐poor groups and visible minorities have higher odds of being in the Unemployment and Unemployment/Less Education clusters (RRR = 1.34–1.76). Youth from Neighborhood dominated by rental accommodation and visible minorities show a lower risk of being in the Unemployment/Less Education/More Social Assistance/More Convictions cluster (RRR = 0.85). Youth from these neighborhoods are less likely to belong to the More Education cluster (RRR = 0.71–0.85).

Furthermore, local labor market conditions negatively influenced individuals' decision to dropout of secondary school (OR: 0.133, *p *> 0.01), thereby contributing to early school leaving and subsequent unemployment (von Simson 2015).

Eight studies illustrate the complexity of how factors such as socioeconomic status, immigration background, educational attainment, and mental health problems intersect with geographical location to influence NEET status (Brattbakk and Wessel 2013; Goldman‐Mellor et al. 2016; Gustafsson, Katz, and Österberg 2017; Helgesson et al. 2018; Magdalene Karyda and Jenkins 2018; Magdalini Karyda 2020; Lallukka et al. 2019; Myhr et al. 2017).

## 4 Discussion

This review systematically explored the intricate relationship between geographical factors and the propensity of young Europeans to become NEETs, uncovering several critical dimensions through which place and space significantly affect youth trajectories. Our findings delineate a multifaceted landscape where geographical disparities not only reflect but also amplify social and economic exclusions, with profound implications for young individuals' access to education and employment opportunities. As this is a scoping review, we did not include an assessment of the methodological quality of the studies reviewed. Therefore, we are unable to draw conclusions regarding the strength of the associations presented in the results section and Table 1 (Pollock et al. 2023). Later in this section, we will discuss the diverse methodologies used in the included studies and provide our perspective on the implications of these methodological variations. Below are the main findings derived in using an inductive approach:

1. Urban‐Rural Divide: Studies included in this review highlights a pronounced urban‐rural divide, where young people in rural areas face heightened risks of becoming NEETs due to limited access to educational institutions and employment opportunities (Grinevica and Rivza 2015; Helgesson et al. 2018; Myhr et al. 2017). This disparity underscores the role of spatial accessibility in shaping life chances, aligning with previous studies that emphasize the rural youth's disadvantage in transitioning to work or higher education (OECD 2018).
2. Neighborhood Effects: The impact of residing in disadvantaged neighborhoods emerged as a pivotal factor influencing NEET status (Brännström and Rojas 2012; Goldman‐Mellor et al. 2016; Magdalene Karyda and Jenkins 2018; Magdalini Karyda 2020). Characteristics of these neighborhoods, such as high crime rates, low educational attainment levels, and poor infrastructure, contribute to a milieu where youth are more likely to disengage from both education and the labor market. These findings resonate with the broader literature on neighborhood effects, which documents how sociospatial environments can entrench cycles of poverty and exclusion (Sampson, Morenoff, and Gannon‐Rowley 2002).
3. Local Labor Market Conditions: Local labor market conditions, including the availability of jobs and the nature of employment opportunities, were found to be crucial in determining the likelihood of youth becoming NEETs (von Simson 2015). In regions with high unemployment rates and limited job prospects, young people encounter substantial barriers to entering the workforce, a challenge that is exacerbated for those without higher education qualifications (Eurofound 2016; Mashcerini and Ledermeier 2017).
4. Sociogeographical dynamics: This review further elucidates the complex sociogeographical dynamics at play, revealing how factors such as socioeconomic status, immigration background, and educational attainment intersect with geographical location to influence NEET status (Brattbakk and Wessel 2013; Goldman‐Mellor et al. 2016; Gustafsson, Katz, and Österberg 2017; Helgesson et al. 2018; Karyda and Jenkins 2018; Magdalini Karyda 2020; Lallukka et al. 2019; Myhr et al. 2017). This aligns with the concept of spatial inequality, which suggests that socioeconomic disparities are deeply entrenched in the geographical landscape, affecting individuals' opportunities based on where they live (Dorling 2015).

The synthesis of the present systematic review with existing scholarly works elucidates a deeper understanding of the geographical disparities affecting NEET status among young Europeans. An understanding that enriching the dialog in the field of youth disengagement and spatial inequalities is likely generalizable to other regions and countries. However, it's also conceivable that cultural factors could have a significant influence.

The evidence of an urban‐rural divide and the detrimental impacts of residing in disadvantaged neighborhoods resonate with prior studies that have highlighted the critical role of spatial accessibility to education and employment opportunities. This confluence of findings underscores the importance of geographical context in shaping young people's life chances, as supported by Eurofound and Chetty et al. who advocate for a nuanced appreciation of spatial determinants in addressing youth opportunities (Chetty, Hendren, and Katz 2016; Eurofound 2023). Our review's insights into local labor market conditions and their influence on youth engagement echo (Bell and Blanchflower 2011) advocacy for regional economic development as a cornerstone of effective youth policy. Such alignment with existing research not only validates our findings but also strengthens the call for policy interventions tailored to the specificities of geographical and regional contexts.

The imperative for tailored educational initiatives emerges as a critical policy recommendation from our review. The disparity in educational access and attainment necessitates investments in digital learning platforms and infrastructural enhancements, especially in rural and disadvantaged urban areas, to bridge the educational divide—a strategy that has seen success in Scandinavia through innovative digital education models (OECD 2015). Concurrently, the localized employment schemes, inspired by the German dual education system, highlight the potential of apprenticeships and entrepreneurship support tailored to local industries and economic contexts, offering pathways for youth engagement and job creation (Euler 2013). Moreover, the neighborhood effects on NEET status advocate for a comprehensive approach to community and urban development. A recent systematic review found that certain interventions, such as obtaining a driver's license or securing independent housing, increased the likelihood of employment for young people in NEET. However, the review also noted that very few interventions are scientifically evaluated and reported (Stea et al. 2024). Strategies that integrate youth services, recreational facilities, and crime reduction programs within urban planning efforts can significantly alter the trajectory for young individuals in disadvantaged neighborhoods, transforming these areas into supportive environments conducive to their development.

### 4.1 Variability in Statistical Methods and Factor Roles in NEET Risk Studies. Implications for Interpretation and Comparisons

The studies examining the impact of geographic factors on NEET status have utilized various roles for factors—confounders, mediators, and dependent/independent variables. These roles influence how results are interpreted and make direct comparisons across studies more challenging. Some studies treat geographic and individual factors as confounders, adjusting for variables like crime rates, ethnicity, and family background to isolate the effect of neighborhood deprivation on NEET risk (e.g., Karyda 2020). While this approach helps control for extraneous variables, it assumes that all relevant confounders have been identified. Missing or misclassified confounders could lead to biased conclusions. Other studies use mediators, which explain the mechanisms linking neighborhood characteristics to NEET outcomes. For instance, Lallukka et al. (2019) suggested that health behavior and education mediate the impact of neighborhood deprivation on NEET status. While this approach attempts to uncover how geographical factors influence NEET risk, misidentifying mediators can obscure true causal pathways. In some studies, geographic and individual factors are treated as dependent or independent variables (e.g., Brattbakk and Wessel 2013), simplifying the analysis. For example, neighborhood characteristics like unemployment rates were treated as independent variables, while individual factors like educational attainment were dependent variables. However, this may overlook the complex interactions between factors and their potential bidirectional relationship. The different ways in which factors are used complicate comparisons across studies. Studies that focus on confounders may underestimate the complexity of the relationship between geography and NEET outcomes, while those focusing on mediators might reveal more intricate causal pathways. This diversity in methodological approaches underscores the challenges of drawing universal conclusions about how geographic factors influence NEET risk. A more integrated approach that considers all three roles could provide a clearer and more comprehensive understanding of the issue.

Moreover, studies investigating NEET risk have employed varied statistical methods to estimate risk, each with its own strengths and limitations. Propensity score matching as used by Karyda (2020), balances the distribution of covariants between the comparative groups, reducing selection bias. However, the effectiveness depends on accurate matching variables, and if important confounders are omitted, results may still be biased. Cumulative risk and probability models, such as those used by Lallukka et al. (2019), estimate the likelihood of NEET status over time, accounting for multiple risk factors. While these models can provide insights into the compounding effect of risk factors, they can become complex and may oversimplify causal relationships by focusing on aggregate probabilities rather than individual trajectories. Matched group designs, such as those employed by Helgesson et al. (2018), compare individuals with similar characteristics but different exposures, helping to control for confounding factors. However, this approach may miss unobserved interactions and lead to reduced statistical power, limiting the generalizability of findings.

These variations in statistical methods contribute to divergent conclusions about NEET risk, complicating comparisons across studies. Propensity score matching may control for observed confounders but not unmeasured ones, cumulative risk models may overemphasize the cumulative effect of individual factors, and matched groups analysis could miss broader risk patterns. The differences in methods highlight the importance of understanding the strengths and limitations of each approach when interpreting results.

### 4.2 Implications of Different Analytical Models in NEET Risk Studies

In addition, the use of group‐based trajectory (GBT) models by Helgesson et al. (2018) and the Quintuple Helix model by Grinevica and Rivza (2015) further complicate comparisons. GBT models categorize individuals into subgroups based on patterns of work disability and unemployment, offering insights into how these patterns evolve (Helgesson et al. 2018). However, this approach may oversimplify the diversity of experiences within the NEET group if key trajectories are overlooked. The Quintuple Helix model, by contrast, considers the interactions between five sectors—political system, economic system, education system, media‐based, and culture‐based public, and the natural environment—emphasizing the societal and environmental factors influencing NEET outcomes (Grinevica and Rivza 2015). While this model broadens the view of NEET risk, its complexity makes it difficult to isolate the specific impact of each sector. Both models provide useful insights—GBT models focus on individual trajectories, while the Quintuple Helix model addresses broader societal dynamics. However, the differences in scope and complexity between these models highlight the challenges of comparing results across studies. A more integrated approach that considers both individual and societal factors could offer a clearer understanding of NEET risk. These methodological differences underline the importance of critically assessing the strengths and limitations of each approach when interpreting results. Given the diversity of methods used in studies on geographical factors affecting NEET risk, it is essential to understand the underlying assumptions of each approach to draw robust conclusions about the factors contributing to NEET status.

Future research should aim to address the complex interplay between geographical disparities and social inequalities that contribute to the NEET phenomenon. Specifically, there is a need for more nuanced studies that explore the impact of digitalization and the global economy on rural and urban divides, particularly in the context of postpandemic recovery (OECD 2020). Longitudinal studies could provide deeper insights into the long‐term effects of geographical location on NEET status, while comparative research across different European regions could uncover the efficacy of localized interventions. Additionally, qualitative research that captures the lived experiences of NEET youth can enrich our understanding of the barriers and opportunities they face, offering a more holistic view of the issue. Bridging these gaps will not only enhance our theoretical understanding but also inform more effective, context‐specific policy interventions.

### 4.3 Strengths and Limitations

Although this review has been conducted diligently, it is important to acknowledge several limitations. First, despite our thorough efforts to systematically locate and categorize studies, there is still a possibility of missing relevant research due to the selection of search terms and databases. Second, the broad range of outcomes assessed, including unemployment, disability pensioning, NEET status, and education dropout, adds complexity to understanding the precise influence of physical variables on each outcome. Third, while not assessing study quality allows for the inclusion of a wider variety of sources, it also means that the findings should be interpreted with caution. The lack of quality assessment may limit the ability to draw firm conclusions about the strength of the evidence presented, and readers should be aware of this when considering the implications of the review Fourth, although all but one of the studies are cohort studies, there remains high heterogeneity in methods, populations, and findings across the studies. This suggests that future research should focus on how variations in study design, geographic focus, and population characteristics influence the overall synthesis. A more in‐depth examination of how these factors impact outcomes would provide a clearer and more nuanced understanding of the evidence baseA strength of the study is that the research team behind the review is interdisciplinary, which we believe has led to a broader literature search and influenced the analysis of the studies. Moreover, our study design precludes the calculation of effect size estimates, emphasizing the need for future research to conduct statistical meta‐analyses to address this gap. Conversely, the review contributes to a comprehensive understanding of an area that remains significantly underexplored in the literature.

## 5 Conclusion

In conclusion, this systematic review has illuminated the significant impact of geographical factors on the NEET status among young people in Europe, uncovering the nuanced ways in which urban‐rural divides, neighborhood characteristics, and local labor market conditions shape youth engagement with education and employment. Through a meticulous analysis of existing literature, this study contributes to a deeper understanding of the spatial dimensions of youth disengagement, offering evidence‐based insights for policymakers, educators, and community leaders.

The findings underscore the urgency of developing targeted interventions that address the root causes of geographical disparities, advocating for inclusive policies that consider the complex sociogeographical dynamics at play. As Europe grapples with the challenges of ensuring equitable opportunities for all its youth, this review calls for a concerted effort to bridge the spatial divide, emphasizing the need for future high quality research to explore innovative solutions that can mitigate the NEET phenomenon. In doing so, we move closer to realizing a society where every young individual has the support and resources necessary to thrive, regardless of their geographical location.

## Ethics Statement

The authors have nothing to report.

## Conflicts of Interest

The authors declare no conflicts of interest.

## Supporting information

Supporting information.

## Data Availability

The search strategies from all databases are shown in the Supporting Information.
