# Supplementary material for: Place Matters: Understanding Geographic Influences on Youth Not in Education, Employment, or Training—A Scoping Review
Source: J Adolesc. 2025 Jan 9;97(3):620–33. doi: 10.1002/jad.12461 (PMC11973842; doi:10.1002/jad.12461)
Supplement: Supplementary file 1 — Supporting information. [file JAD-97-620-s001.docx]

# Appendix 1:

| Database | Interface | Number of references | Date |
| --- | --- | --- | --- |
| Medline | Ovid | 16,146 | 21.02.2023 |
| Embase | Ovid | 16,934 | 21.02.2023 |
| PsycInfo | Ovid | 5,971 | 21.02.2023 |
| Scopus | Elsevier | 12,075 | 21.02.2023 |
| All  After duplicate removal in Covidence |  | 51,126  33,323 |  |

# Embase

Database: Embase <1974 to 2023 January 30> Search Strategy:

--------------------------------------------------------------------------------

1 NEET.ti,ab,kw,kf. (234)

2 (NEETS or Hikikomori*).ti,ab,kw,kf. (270)

3 (Noneducat* or non educat*).ti,ab,kw,kf. or exp disadvantaged population/ (1049)

4 unemploy*.ti,ab,kw,kf. or Unemployment/ (37420)

5 (Socially excluded or truant* or economic* inactiv*).ti,ab,kw,kf. (757)

6 ((No or out or "not" or dropout or disconnect* or attach* or detach*) adj2 (job* or work* or school* or educat* or

employ* or training*)).ti,ab,kw,kf. (103035)

7 (youth* or adolescen* or juvenile* or early adulthood).ti,ab,kw,kf. (630714)

8 (young adj2 (adult* or person* or individual* or people* or population* or man or men or wom#n)).ti,ab,kw,kf.

(306640)

9 Adolescent/ (1718167)

10 Young Adult/ (485970)

11 7 or 8 or 9 or 10 (2423334)

12 1 or 2 or 3 or 4 or 5 or 6 (140206)

13 11 and 12 (22130)

14 review.pt. (3006963)

15 exp "review"/ (3059107)

16 exp controlled clinical trial/ (952153)

17 14 or 15 or 16 (4237273)

18 ((opportunit* or disconnect*) adj1 (youth or adolescent* or juvenile* or early adulthood or (young adj2 (adult*

or person* or individual* or people* or population* or man or men or wom#n)))).ti,ab,kw,kf. (139)

19 13 or 18 (22249)

20 19 not 17 (20148)

21 limit 20 to (yr="2000 -Current" and (danish or english or norwegian or swedish)) (16934)

# PsycInfo

Database: APA PsycInfo <1806 to January Week 4 2023> Search Strategy:

--------------------------------------------------------------------------------

1 NEET.ti,ab. (159)

2 (NEETS or Hikikomori*).ti,ab. (212)

3 (Noneducat* or non educat*).ti,ab. (326)

4 unemploy*.ti,ab. (16738)

5 exp Unemployment/ or exp Disadvantaged/ (13439)

6 (Socially excluded or truant* or economic* inactiv*).ti,ab. (957)

7 ((No or out or "not" or dropout or disconnect* or attach* or detach*) adj2 (job* or work* or school* or educat* or

employ* or training*)).ti,ab. (52093)

8 (youth* or adolescen* or juvenile* or early adulthood).ti,ab. (352575)

9 (young adj2 (adult* or person* or individual* or people* or population* or man or men or wom#n)).ti,ab. (112803)

10 exp Adolescent Development/ (64631)

11 1 or 2 or 3 or 4 or 5 or 6 or 7 (77784)

12 exp Disadvantaged/ (8566)

13 11 or 12 (77784)

14 8 or 9 or 10 (428618)

15 13 and 14 (11137)

16 exp "Systematic Review"/ or exp "Literature Review"/ (23718)

17 exp clinical trials/ (13446)

18 ((opportunit* or disconnect*) adj1 (youth or adolescent* or juvenile* or early adulthood or (young adj2 (adult*

or person* or individual* or people* or population* or man or men or wom#n)))).ti,ab. (187)

19 15 or 18 (11286)

20 16 or 17 (37087)

21 19 not 20 (11246)

22 limit 21 to ((danish or english or norwegian or swedish) and yr="2000 -Current") (8381)

23 limit 22 to ("0100 journal" or "0110 peer-reviewed journal" or "0120 non-peer-reviewed journal" or "0130 peer-reviewed status unknown" or "0500 electronic collection") (5971)

# Medline

Database: Ovid MEDLINE(R) and Epub Ahead of Print, In-Process, In-Data-Review & Other Non-Indexed Citations and Daily

<1946 to January 30, 2023>

Search Strategy:

--------------------------------------------------------------------------------

1 NEET.ti,ab,kw,kf. (206)

2 (NEETS or Hikikomori*).ti,ab,kw,kf. (200)

3 (Noneducat* or non educat*).ti,ab,kw,kf. (344)

4 unemploy*.ti,ab,kw,kf. or Unemployment/ (25554)

5 (Socially excluded or truant* or economic* inactiv*).ti,ab,kw,kf. (643)

6 ((No or out or "not" or dropout or disconnect* or attach* or detach*) adj2 (job* or work* or school* or educat* or

employ* or training*)).ti,ab,kw,kf. (78529)

7 (youth* or adolescen* or juvenile* or early adulthood).ti,ab,kw,kf. (508075)

8 (young adj2 (adult* or person* or individual* or people* or population* or man or men or wom#n)).ti,ab,kw,kf.

(231432)

9 Adolescent/ (2200530)

10 Young Adult/ (1003568)

11 7 or 8 or 9 or 10 (2943862)

12 1 or 2 or 3 or 4 or 5 or 6 (103646)

13 11 and 12 (22968)

14 review.pt. or exp "review"/ (3121840)

15 systematic review.pt. or exp "systematic review"/ (218609)

16 controlled clinical trial.pt. or exp controlled clinical trial/ (677259)

17 14 or 15 or 16 (3896228)

18 ((opportunit* or disconnect*) adj1 (youth or adolescent* or juvenile* or early adulthood or (young adj2 (adult*

or person* or individual* or people* or population* or man or men or wom#n)))).ti,ab,kw,kf. (113)

19 13 or 18 (23060)

20 19 not 17 (20904)

21 limit 20 to (yr="2000 -Current" and (danish or english or norwegian or swedish)) (16146)

# Scopus

PUBYEAR > 1999 AND ( ( ( ( TITLE-ABS ( neet OR neets OR Hikikomori* OR noneducat* OR "non educat*" OR unemploy* OR "Socially excluded" OR truant* OR "economic* inactiv*" ) ) OR ( TITLE-ABS ( ( ( no OR out OR "not" OR dropout OR disconnect* OR attach* OR detach* ) PRE/2 ( job* OR work* OR school* OR educat* OR employ* OR training* ) ) ) ) ) AND ( ( TITLE-ABS ( ( young W/2 ( adult* OR person* OR individual* OR people* OR population* OR man OR men OR wom#n ) ) ) ) OR ( TITLE-ABS ( youth* OR adolescen* OR juvenile* OR "early adulthood" ) ) ) ) OR ( TITLE-ABS ( ( opportunit* OR disconnect* ) W/1 ( youth OR adolescent* OR juvenile* OR "early adulthood" OR ( young W/2 ( adult* OR person* OR individual* OR people* OR population* OR man OR men OR wom#n ) ) ) ) ) ) AND NOT ( TITLE ( review* OR trial* ) ) AND ( EXCLUDE ( DOCTYPE , "re" ) OR EXCLUDE ( DOCTYPE , "ch" ) OR EXCLUDE ( DOCTYPE , "bk" ) OR EXCLUDE ( DOCTYPE , "cr" ) ) AND ( LIMIT-TO ( LANGUAGE , "English" ) OR LIMIT-TO ( LANGUAGE , "Danish" ) OR LIMIT-TO ( LANGUAGE , "Norwegian" ) OR LIMIT-TO ( LANGUAGE , "Swedish" ) )

# Updated search: 15.01.2024

| Database | Interface | Number of references | Date |
| --- | --- | --- | --- |
| Medline | Ovid | 779 | 15.01.2024 |
| Embase | Ovid | 1,443 | 15.01.2024 |
| PsycInfo | Ovid | 200 | 15.01.2024 |
| Scopus | Elsevier | 1,243 | 15.01.2024 |
| All  After duplicate removal in Covidence |  | 3,665  2,535 |  |
